# Design of a dual randomized trial in a type 2 hybrid effectiveness—implementation study

**DOI:** 10.1186/s13012-023-01317-9

**Published:** 2023-11-23

**Authors:** June Stevens, Sarah Denton Mills, Mary-Louise Millett, Feng-Chang Lin, Jennifer Leeman

**Affiliations:** 1 Departments of Nutrition and Epidemiology, Gillings School of Global Public Health, University of North Carolina at Chapel Hill, Chapel Hill, NC 27599 USA; 2 Lineberger Comprehensive Cancer Center, Department of Health Behavior, Gillings School of Global Public Health, University of North Carolina at Chapel Hill, Chapel Hill, USA; 3 UTHealth Houston Institute for Implementation Science, University of Texas Health Science Center at Houston, Houston, TX 77030 USA; 4 Department of Biostatistics, Gillings School of Global Public Health, University of North Carolina at Chapel Hill, Chapel Hill, NC 27599 USA; 5 School of Nursing, University of North Carolina at Chapel Hill, Chapel Hill, NC 27599 USA

**Keywords:** Randomized trial, Methods, Implementation science

## Abstract

**Background:**

Dual randomized controlled trials (DRCT) are type 2 hybrid studies that include two randomized trials: one testing implementation strategies and one testing an intervention. We argue that this study design offers efficiency by providing rigorous investigation of both implementation and intervention in one study and has potential to accelerate generation of the evidence needed to translate interventions that work into real-world practice. Nevertheless, studies using this design are rare in the literature.

**Main text:**

We construct a paradigm that breaks down the components of the DRCT and provide a step-by-step explanation of features of the design and recommendations for use. A clear distinction is made between the dual strands that test the implementation versus the intervention, and a minimum of three randomized arms is advocated. We suggest an active treatment arm that includes both the implementation strategy and intervention that are hypothesized to be superior. We suggest two comparison/control arms: one to test the implementation strategy and the second to test the intervention. Further, we recommend selection criteria for the two control arms that place emphasis on maximizing the utility of the study design to advance public health practice.

**Conclusions:**

On the surface, the design of a DRCT can appear simple, but actual application is complex. We believe it is that complexity that has limited its use in the literature. We hope that this paper will give both implementation scientists and trialists who are not familiar with implementation science a better understanding of the DRCT design and encouragement to use it.

**Supplementary Information:**

The online version contains supplementary material available at 10.1186/s13012-023-01317-9.

Contributions to the literature

- We describe dual randomized controlled trials (DRCT), a design for use in type 2 hybrid studies that provides a rigorous test of both the implementation strategy and the intervention in one study to accelerate uptake of improved health practices.
- Our review of 6 years of publications from four implementation science-focused journals found no studies with published results from a DRCT.
- We provide a paradigm to guide investigators through the process of designing a DRCT.
- The paradigm has the potential to increase the use of DRCT designs and promote generation of evidence needed to shift interventions that work into real-world practice.

## Background

In 2012, Curran et al. described three types of implementation studies differentiated by their relative emphasis on the implementation strategies and the intervention: type 1 focuses on relationships between interventions and health outcomes, type 3 focuses on relationships between implementation strategies and implementation outcomes, and type 2 focuses on both [1]. In their 2022 paper [2], which reflected on a decade of use of hybrid studies, the authors comment that in the original conception of hybrid types, it was assumed that a randomized controlled trial (RCT) would be included in each hybrid study and that in a hybrid type 2, there could be multiple randomizations. In the more recent paper [2], they revised that stance and do not require the use of any particular study design. Our paper addresses the subset of type 2 hybrid studies that include two randomized controlled trials (RCTs): one testing implementation strategies on implementation outcomes and one testing an intervention on health-related outcomes. We call this study design a “dual randomized controlled trial (DRCT)” following the “dual strands” terminology utilized in the Standards for Reporting Implementation Studies (StaRI) checklist table [3] in which the two strands are shown as one column describing a sub-study of implementation strategies and a second column describing a sub-study of the health intervention that is implemented.

Type 2 hybrid DRCTs offer efficiency by including investigation of both the implementation and intervention in one study, creating the potential to accelerate the generation of the evidence needed to translate interventions that work into real-world practice. Nevertheless, in our review of 6 years of publications from four implementation science-focused journals (*Implementation Science, Contemporary Clinical Trials, Translational Behavioral Medicine, and Implementation Research and Practice*), we found only 34 studies (see Additional file 1) in which the authors reported using a type 2 hybrid study. None of those studies met the definition shown above for a DRCT, i.e., showed results from randomized groups in an RCT comparing both the effect of an implementation strategy on an implementation outcome and the effect of an intervention on a health-related outcome. Three of the publications [4–6] described study protocols that, if enacted as described, would meet those criteria, but a broad search of the literature did not find corresponding publications showing results. Two publications [7, 8] showed qualitative findings, but no tests of hypotheses deriving from the randomized trials. Another study [9] used a factorial design and a second randomization that could lead to correlations in the data that were not accounted for in the analysis and likely resulted in an incorrect estimate. Curran et al. [2], cite low feasibility and lack of resources as reasons for little use of designs that test both implementation strategies and interventions in a randomized trial; however, we believe another factor that reduces their use is confusion surrounding study construction.

To address that confusion, we present a deconstruction of the DRCT design. Starting with the classic RCT design, we suggest a paradigm that breaks down the components of a DRCT and discuss matters specific to study arm formation. We use illustrative examples to help clarify concepts. It is our hope that the paradigm described here will unravel some of the complexity and encourage investigators to use the DRCT design to advance research that can improve health and reduce health disparities.

## Main text

### The classic RCT

In the classic two-arm RCT design, following recruitment, determination of eligibility, and measurement of baseline variables, participants or groups are randomized to active or control study arms. In the text below, we emphasize cluster-randomized trials, but whether individuals or groups are randomized, the general study design is the same, and we do not dwell on that difference here. An intervention is delivered to the active study arm, and the control arm receives either a comparison intervention, a placebo attention control, or no new intervention. The outcome is subsequently measured in both arms after a pre-determined time interval, and the difference in the outcome between study arms is calculated to form the estimand [10, 11].

**Fig. 1:**
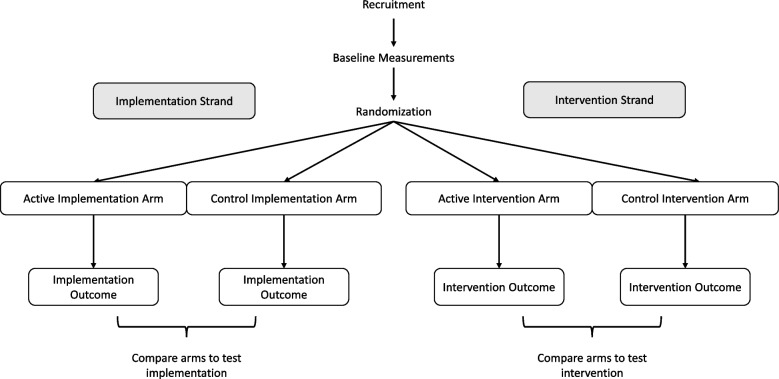
Type 2 hybrid study using DRCT with four arms

Initially, it may be helpful to envision a DRCT as two classic RCTs occurring side-by-side with overlapping recruitment and randomization steps (Fig. 1). The effect of implementation strategies on implementation outcomes is tested in one RCT, and in a second RCT, the effect of the intervention on health-related outcomes is tested. In practice, often multiple implementation strategies and intervention components are delivered at different levels. Each RCT would (usually) have a single hypothesis identified as primary, following the Consolidated Standards of Reporting Trails (CONSORT) guidelines [12]. Thus, in a DRCT, there are two primary hypotheses that are equally valued and tested in a single study that includes adequate power for both of the primary statistical models. Whether to apply correction for multiple testing in this situation is controversial and will not be discussed here [12–14]. Following usual trial methodology [12], the two primary statistical models are fully pre-specified before the beginning of the study, which is often demarcated by the initiation of randomization. Numerous secondary hypotheses are also studied.

Rather than focusing on either the intervention or the implementation strategy, the hybrid type 2 study spreads available resources to study both. To justify doing so, Curran et al. [1] recommended specific conditions for the conduct of a hybrid type 2 study; however, they do not give specific guidance for the use of a DRCT design. A DRCT is used when investigators want rigorous evidence that has low risk of bias and quantifiable certainty indicating whether or not there are differences in implementation and health-related outcomes among two or more alternative implementation and intervention approaches that are in equipoise.

Curran et al. [2] note that the simultaneous examination of implementation strategies and interventions in a type 2 study is sometimes best served by a liberal approach that does not involve an adequately powered randomized design. We agree that type 2 studies using more liberals designs are important to the field; however, we do not classify a DRCT as a liberal design, as it stands at the opposite end of the spectrum of type 2 studies. Adherence to the tenets of an RCT (including a DRCT) requires pre-specification of intentions, masking, and reduced flexibility to make changes in the protocol and/or procedures once the trial begins [3, 12, 15]. Therefore, other designs are usually a better choice for exploratory or demonstration hybrid type 2 studies.

Examples of common implementation outcomes are reach to individual participants (number, %, representativeness), fidelity of intervention delivery, cost to implement, individual adoption of the intervention, institutional adoption of the intervention, and maintenance over time [16]. Intervention outcomes are health behaviors, health outcomes, and health-related environmental characteristics. Respective examples include smoking, incident stroke, and healthy food availability. These outcomes are often studied as counts, proportions, incidence, or means at the end of the study period or as changes from baseline. Here we assume superiority trials are being used; however, a DRCT can be a non-inferiority trial [15]. For example, a new implementation and intervention could be hypothesized to produce effects that are not inferior to an existing treatment that requires more resources.

### Exposures and study arms

Figure 1 is familiar to investigators experienced in RCT designs, but it is not an ideal depiction of a type 2 hybrid DRCT because in that design each study arm is, by definition, assigned an implementation exposure and an intervention exposure. Here, we use the word “exposure” following the text by Rothman et al. [17] that defines the term broadly as a potential causal factor that can be a behavior, treatment, trait, or exogenous factor. An exposure can be naturally occurring or randomized to be part of the study arms of an RCT (as in this paper).

In Table 1, we introduce notation to represent five types of exposures that can be paired to produce a DRCT design with three study arms. More arms are possible, but at least three are required. In this paper, we focus on the three-arm design. We use “S” to indicate implementation strategy exposures and “P” to indicate intervention exposures with reference to the 7 Ps listed by Brown et al. [18] to describe different categories of interventions found in dissemination and implementation research (programs, practices, principles, procedures, products, pills, and policies). We use the words strategy (S) and program (P), rather than implementation and intervention, to differentiate exposures from arms and to escape the confusion caused by the common first letters and strong alliteration in the words implementation and intervention. Active and control exposures are denoted by “a” and “c,” respectively. The implementation strategy exposure used in the control arm (SxPc) for the test of the intervention is denoted by “Sx” rather than “Sc” to indicate that it is not the same as in the control arm for the test of the implementation (ScPa).

**Table 1:** Five exposures that compose 3 study arms

5 types of study exposures	3 study arms that are randomized
***Pa*** (active intervention program): an evidence-based intervention hypothesized to have a stronger effect on the health outcome compared to the control. It is used in both active and control arms in the test of implementation strategies ***Pc*** (control for active intervention program): an intervention that can be comparison treatment, an attention control or usual care/no new activity ***Sa*** (active implementation strategies): implementation strategies hypothesized to have a stronger effect on implementation outcomes compared to the control. It is used in active arm of test of implementation strategies ***Sc*** (control for active implementation strategies): implementation strategies hypothesized to have a measurable non-zero effect on the implementation outcome. It is used in control arm in test of implementation strategies ***Sx*** (implementation strategies used to administer control intervention program): the implementation strategies are designed to be consistent with the type of control intervention	***SaPa***: active arm in test of implementation strategies and intervention program ***ScPa***: control arm in test of implementation strategies ***SxPc***: control arm in test of intervention program

Although the assignment of pairs of exposures to study arms has similarities to a factorial design, that paradigm is not a good fit for a DRCT. A conventional 2 × 2 factorial design has two levels of one factor (e.g., high or low education) applied to two levels of another (e.g., young or old age) to form four experimental sets. The factors that make up the four pairs are the same (age and education) and there is only one outcome (e.g., body weight) and the factor effect can be derived from the difference between factor levels. A DRCT studies two primary outcomes: one related to implementation and one related to health. Also, the four experimental sets are not always, or even usually, composed of the same two levels of the two factors defined in the same way, and the investigators are not usually interested in the average implementation effect in groups that received either the active or the control intervention.

Core function and form, two key constructs used to describe complex health interventions [19, 20], can also be applied to implementation strategies. Core function is the fundamental purpose or desired effect (on patients, health professionals, or staff) of a set of activities hypothesized to support change. An implementation example is education to establish the knowledge base necessary for community health workers to deliver a specific intervention. The form describes how activities and other supports operationalize the core functions of the intended implementation. For example, education may take the form of hard copy materials, online videos, and/or one-to-one or group-based in-person teaching. The form can vary in dose (frequency and duration). The core functions will influence content. One or more aspects of core functions, form, and/or content will differ between the active and comparison exposures, and the core functions and content of the implementation strategies serve the intervention within a study arm.

#### Implementation strategies and the intervention are linked and theoretically delivered in sequence

Ideally, implementation strategies are constructed by identifying multilevel determinants that are facilitators and barriers to the successful uptake of the intervention in the target population. Thus, the implementation strategies are relevant to the core functions, form, and other characteristics of the intervention. The determinants identified are used to create implementation strategies that are hypothesized to support the reach, adoption, fidelity, and/or sustainment of the intervention [21]. Often an implementation strategy is applied at the provider level and enhances the ability of the provider to deliver the intervention to participants. The nature of this association sets up a natural order in which the implementation strategies theoretically, if not operationally, precede the intervention.

#### SaPa is the active arm that investigators want to evaluate as a candidate for dissemination, spread and/or scaling up

**Fig. 2:**
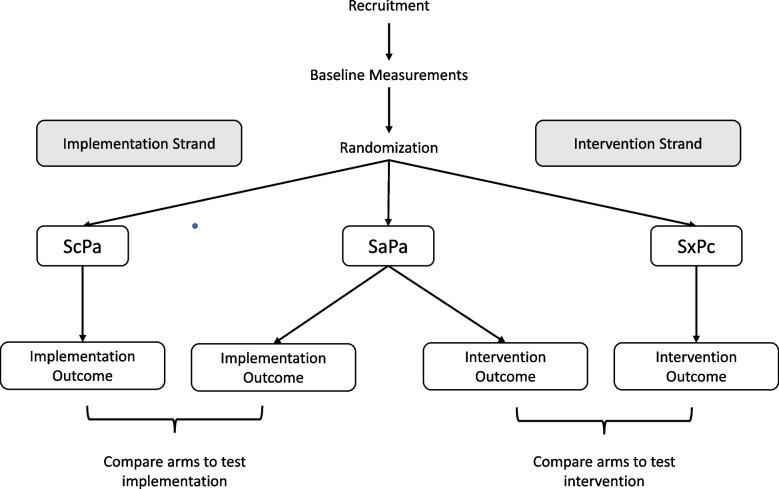
Type 2 hybrid study using DRCT with three arms

Here we assume that the investigators are interested in the usefulness of a newly developed implementation exposure (Sa) applied to a specific intervention program (Pa). The intervention can be untested, but in implementation science, often the intervention is evidence-based. A DRCT is conducted with the ultimate sustainment and spread of *SaPa* in mind if it is proven to produce stronger effects on intervention uptake and the health outcome than the control conditions. Therefore, the three-arm design in Fig. 2 shows *SaPa* in the center of the diagram serving as the active study arm in the test of both the implementation strategy and the intervention against their respective control arms. A DRCT is formed by randomizing units of the target sample (be they individuals, providers, or institutions) to one of these three arms to construct three groups.

### Example of active implementation strategies and intervention program (SaPa) in a hybrid type 2 DRCT examining implementation uptake and health effects

Below we walk the reader through the DRCT design using constructed examples that evaluate acceptance and commitment therapy (ACT) [22] as the active intervention program (Pa). ACT is an existing evidence-based treatment (i.e., intervention) for anxiety. The target population selected for the implementation strategy is therapists in clinics, and the target population for the intervention is adolescent patients with anxiety disorder, a population for whom evidence of ACT efficacy is emerging [23]. The active implementation strategy (Sa) is clinical supervision in a 1-h group meeting facilitated by a therapist with expertise in ACT. Over a 6-month period, therapists at the clinic meet biweekly, in-person to share situations encountered and lessons learned with each other and to get expert input on strategies for overcoming challenges that arise in the patients they are treating using ACT. These activities are in addition to the six, 20-min online training sessions of didactic content on ACT for therapists that were already being provided at all the clinics. The unit of randomization is the clinic. The primary implementation outcome variable is the percentage of eligible patients that complete at least 8 sessions of the 10-session ACT intervention. The primary intervention outcome variable is the percent change from baseline in patients’ reported anxiety symptoms on a standardized, interviewer-administered questionnaire. As recommended in the CONSORT guidelines [12], outcome variables are collected by research staff who are not implementation or intervention providers and are masked to randomization assignments when feasible.

### Example of comparison of implementation outcomes in the active versus control implementation arms

The control implementation arm will include the same intervention exposure (ACT) as described above for the active implementation arm, but will have a different implementation exposure added to the pre-existing six, 20-min online sessions. The control implementation exposure (Sc) in the control arm (ScPa) could be a time-matched alternative in the form of group meetings for therapists focused on business development for the practice. Like the active arm, therapists at the clinic meet biweekly in-person, but the topics discussed are focused on issues such as networking and securing additional patients. The comparison of the percentage of eligible patients who have completed at least 8 sessions of the 10-session ACT intervention in the *SaPa* and *ScPa* arms is the estimand [10, 11]. This design holds the intervention constant while comparing two different implementation strategies. It is the same design commonly used to test the primary hypothesis in a hybrid type 3 trial [24, 25] and forms one of the two strands of a DRCT.

Features and recommendations for the study of the implementation:

1. *Using the same intervention exposure across arms*. Keeping the intervention exposure constant enhances the interpretation of differences in the impact of the active versus the control implementation strategy on implementation outcomes. Nevertheless, in behavioral work, even when the same intervention is intended differences can occur across arms. For example, the fidelity of the intervention may be enhanced as a result of an active implementation strategy that creates better-trained providers who are more effective in the delivery of the intervention. In this example, enhanced fidelity caused by the implementation could mediate effects of the intervention on the health outcome. In studies in which investigators hypothesize improvement of the intervention by the implementation, it will be a priority to examine the impact of the different implementation strategies on the health outcome. Although very important, that analysis is not a requirement in a DRCT. It is an additional test of the implementation strategies, not the randomized test of the intervention.
2. *Selecting implementation strategies for the active (Sa) and control (Sc) exposures to construct a question of strong scientific merit.* Investigators may want to compare two sets of implementation strategies newly introduced to a sample or a newly introduced set of implementation strategies compared to strategies that already exist in the sample (usual care). If the research seeks to improve or expand on the implementation strategies that are already in place in the study setting, then usual care is an option that can provide the control implementation strategy (Sc) to be compared with alternative or additional implementation strategies (Sa). This circumstance brings up the often-ignored point that evidence-based intervention programs already include some sort of implementation strategy in order for the intervention to have taken place previously. New hybrid research may be launched when it is determined that the implementation strategies included in the evidence-based intervention program are not adequately promoting uptake of the intervention or need to be adjusted to serve a different setting. For instance, the Kaiser Permanente bundle [26], which aims to control hypertension, includes multiple implementation strategies (e.g., follow-up visits for blood pressure management, education of healthcare staff), but new implementation strategies (e.g., delivery of a toolkit with additional training and education materials) added to those can be tested [27]. In hybrid research those implementation strategies that are part of the evidence-based intervention program are usually maintained in both study arms and not the subject of study.

A distinguishing attribute of implementation research is that if the usual care and/or new implementation exposures are known to likely have no effect on the implementation outcomes, the information gained from the research is often limited. For example, if we assume a situation in which there is no mention of ACT, or use of ACT existing in clinics prior to the research, then a usual care exposure in the implementation control arm would likely result in therapists having no increase in awareness or knowledge of the ACT intervention, and their preparation for and delivery of the ACT program would be zero (for all practical purposes). Collecting measurements on such an implementation with a zero outcome is not valuable as the outcome is essentially already known. In addition, comparisons of zero implementation endpoints in the control arm to those in an active implementation arm may be statistically significant, even if unacceptably weak. Under most circumstances, this type of implementation control group should be avoided in favor of a control that supports equipoise and provides stronger public health relevance.

### Comparison of health outcomes in the active and control intervention arms

The goal in this strand of the DRCT is to determine if the active intervention, supported using the active implementation strategy, is more effective at improving the health outcome in the target population compared to a control arm. As shown in the figures, the study sample, randomization, and active arm (SaPa) are shared between strands; but the control arm, outcomes, and tests of statistical significance are different and separate as two different hypotheses are being tested. In the study of the health outcome, the decision process of choosing the control arm should not be substantially different from that used outside of implementation research to test an intervention. Investigators choose a control arm to compare to the active arm (SaPa) that maximizes relevance and health impact, given resources. Those criteria should be given prominence over maximizing effect size.

**Fig. 3:**
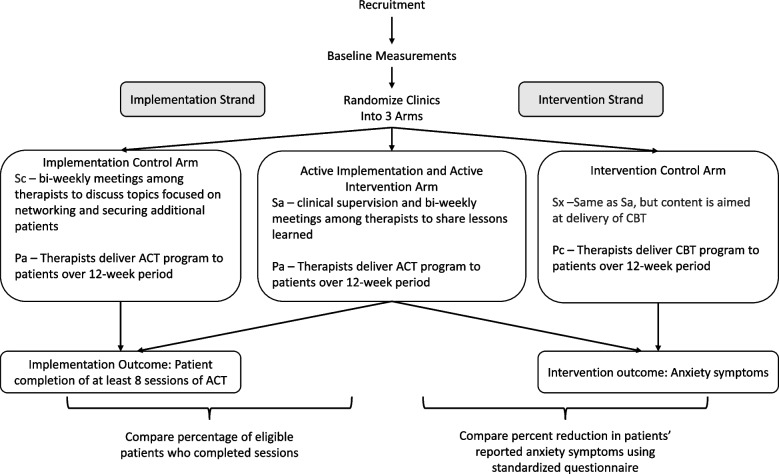
Example hybrid type 2 DRCT examining fidelity of the ACT evidence-based intervention and changes in anxiety symptoms
  ii) An alternative treatment for anxiety that shares few characteristics with ACT such as free membership in a commercial gym.
  iii) A placebo intervention that provides attention but is not hypothesized to substantially impact anxiety such as a book club.
  iv) No intervention activities.

Features and recommendations for the study of the intervention:

1. *Combining effects of the implementation strategies and the intervention on the health outcome*. In this strand of a DRCT, the active implementation strategy seems the natural implementation exposure to use in both intervention arms (comparing SaPa vs SaPc). This design offers the opportunity to hold the implementation strategy constant while contrasting the active and comparison interventions and appears to possess the same logic as the design recommended above for the test of the implementation outcomes. However, this appearance is misleading. It is rarely logical to have the entirely same implementation strategy used to prepare for the administration of two different interventions. The implementation strategies are typically tailored to address barriers and facilitators specific to implementing a particular intervention within the given context. Also, the active and control implementation strategies will need to be consistent with the respective core functions of the change processes that the two interventions seek to cause. As mentioned earlier, theoretically, the application of the implementation precedes the intervention, but it is useful to characterize the intervention before designing the implementation. Regardless of whether the active and control implementations are similar or different, the active implementation will be incorporated into the packaged protocol alongside the intervention should it be disseminated or spread. Therefore, in this strand of the research, it is more relevant for investigators to compare the impact of the “implemented interventions” rather than to attempt to dissect effects on the health outcome.
2. *Examples of different types of control interventions.* Below we list different possible control intervention exposures (Pc) assuming that the active arm delivers ACT to adolescent patients with anxiety disorder (as in our example above):
  i) An alternative treatment for anxiety that has characteristics similar to ACT such as cognitive behavioral therapy (CBT) (as in Fig. 3).

All of these types of control interventions could be new or they could be ongoing in the research setting and, in the latter situation, would be called usual care.

3. *Implementation exposure (Sx) in the control intervention arm (SxPc)*. Below we discuss the selection of implementation strategies to complement the four control intervention examples shown above.
  i) *Implementation exposure when control intervention consists of activities that are similar in function and form to those in the active intervention.* In our first example above, the two interventions to be compared (ACT and CBT) are comprised of similar activities, setting, and staff, but are different in content. Therefore, the implementation strategy involves similar form (clinical supervision and subsequent meetings) and function (peer networking and skills building) but different content (ACT skills vs. CBT skills). In this example, the forms of the implementation as well as the amount of attention (interaction with implementation staff and investigators) given to the therapists during the implementation could be equal in time. This matching would encourage similar uptake and fidelity of the interventions and reduces or avoids the Hawthorne effect [28, 29], which poses that attention alone can change behavior. Although matching the amount of attention to targets closely is preferable, investigators are often satisfied to only approximately match attention or may choose not to address it. An attention-matched design would enhance separation of intervention effects on the health outcome from possible effects of the implementation, although as noted above, that is not a requirement.
  ii) *Implementation exposure when the control intervention consists of components that are not similar in function or form to those in the active intervention.* Investigators could propose to compare the active implementation (Sa) and intervention (Pa) shown in Fig. 3 to a gym membership that provides exposure to a physical activity-rich environment and help (coaching) from gym personnel (Pc). An example implementation would use navigators to distribute free gym memberships. This difference in the intervention (staff delivering ACT in a clinic versus environmental change and coaching in a gym) requires differences in the core functions, form, and content of the implementation strategies. In this example, the investigators would be unable to assume the implementation strategies would have a similar impact on mediators of the health outcome; however, this assumption is not necessary if conclusions are drawn comparing the impact of the health outcomes on the two interventions as implemented.
  iii) *Implementation exposures when the control intervention is a placebo/attention control.* A control intervention can be hypothesized to cause little or no effect on the health outcome, but to provide equal attention to the target sample. A book club could provide such an attention control in our example. The control implementation exposure (Sx) in the control arm (SxPc) could be activities that employ central research staff to support staff local to the clinic in the formation of a book club for clinic patients with anxiety disorder.
  iv) *Sx if the control intervention is usual care/no intervention*. Changes in health-related outcomes in the absence of an intervention have a more useful interpretation than changes in the implementation outcomes when there are no implementation strategies because in the latter situation essentially zero change can be expected (as discussed above). When emphasis is on testing the health outcome, there might be no new intervention activities or implementation strategies introduced. If the control intervention is usual care, there will likely be a usual care implementation already in place, and activities would continue without change. Wait listing is a type of “no intervention” control.
4. *Closing thoughts on choosing the control arm for the intervention*. It may be surprising that selection of the control exposure (Sx) in the control arm (SxPc) of the test of the intervention received so much attention here, but that selection does influence the interpretation of results and can be difficult. It is our hope that the list of options presented in this section will help investigators think through the best choice for their project. The examples shown were meant to facilitate understanding, not to limit investigators in their choice of core functions, forms and content in the implementation and intervention exposures.

## Conclusions

Despite the potential of type 2 hybrid DRCTs to advance the spread of effective behavioral research and improve health, they remain rare in the published literature. We admit that our review of four journals over the last 6 years was incomplete and may have missed studies that were DRCTs but did not use the type 2 hybrid terminology [24]. The goal of our literature review was to find a few example DRCT studies and get a rough idea of how many such studies were being published. We chose a limited number of journals focused on implementation science, translational science or randomized control trials that we thought would be good candidates for finding a range of type 2 hybrid studies. The 34 papers we found that were called hybrid type 2 revealed complicated designs often with large numbers of implementation strategies, intervention components and outcomes. Many used intricate timeframes with repeated measures that were difficult to evaluate. Some maximized effect size in the experimental arm by using an obviously inferior control arm (abandoning equipoise) or had deviations from the guidance provided by the CONSORT Guidelines [12]. Putting those issues aside, when we drilled down into the text to find studies that used RCTs to provide valid tests of both changes in implementation outcomes and intervention outcomes between randomized active and control groups, we found none.

This paper aimed to demystify the DRCT design by deconstructing the exposures applied in the design and considering the nuanced process of defining and combining the elements required. Admittedly application of the deconstruction process required a level of detail that could be called excessive; however, the dearth of published papers using the DRCT design speaks strongly of a need for this type of in-depth explanation. It is our hope that the paradigm laid out here will encourage investigators to make use of DRCT’s to advance the development, sustainment, dissemination, spread, scale up, and impact of implemented interventions that can improve health.

## Supplementary Information

**Additional file 1.** Hybrid type II studies found in literature review. Publications in which the authors call their study a Hybrid type II.

## Data Availability

Not applicable.

## Abbreviations

ACT: Acceptance and commitment therapy
CBT: Cognitive behavioral therapy
CONSORT: Consolidated Standards for Reporting Trials
DRCT: Dual randomized controlled trial
Pa: Active intervention program
Pc: Control for active intervention program
RCT: Randomized controlled trial
Sa: Active implementation strategies
SaPa: Active arm in test of implementation strategies and intervention program
Sc: Control for active implementation strategies
ScPa: Control arm in test of implementation strategies
StaRI: Standards for Reporting Implementation Studies
Sx: Implementation strategies used to administer control intervention program
SxPc: Control arm in test of intervention program
