# Supplementary material for: Design of a dual randomized trial in a type 2 hybrid effectiveness—implementation study
Source: Implement Sci. 2023 Nov 23;18:64. doi: 10.1186/s13012-023-01317-9 (PMC10666326; doi:10.1186/s13012-023-01317-9)
Supplement: Supplementary file 1 — Additional file 1. Hybrid type II studies found in literature review. Publications in which the authors call their study a Hybrid type II. [file 13012_2023_1317_MOESM1_ESM.docx]

Hybrid type 2 studies found in 4 journals over 6 years

1. Aifah AA, Odubela O, Rakhra A, Onakomaiya D, Hu J, Nwaozuru U, et al. Integration of a task strengthening strategy for hypertension management into HIV care in Nigeria: a cluster randomized controlled trial study protocol. Implementation Science. 2021 Nov 16;16(1):96.
2. Berkel C, Smith JD, Bruening MM, Jordan N, Fu E, Mauricio AM, et al. The Family Check- Up 4 Health: Study protocol of a randomized type II hybrid effectiveness– implementation trial in integrated primary care (the healthy communities 4 healthy students study). Contemporary Clinical Trials. 2020 Sep 1;96:106088.
3. Cella D, Garcia SF, Cahue S, Smith JD, Yanez B, Scholtens D, et al. Implementation and evaluation of an expanded electronic health record-integrated bilingual electronic symptom management program across a multi-site Comprehensive Cancer Center: The NU IMPACT protocol. Contemp Clin Trials. 2023 May;128:107171.
4. Chinman M, Daniels K, Smith J, McCarthy S, Medoff D, Peeples A, et al. Provision of peer specialist services in VA patient aligned care teams: protocol for testing a cluster randomized implementation trial. Implementation Science. 2017 May 2;12(1):57.
5. Chinman M, Goldberg R, Daniels K, Muralidharan A, Smith J, McCarthy S, et al. Implementation of peer specialist services in VA primary care: a cluster randomized trial on the impact of external facilitation. Implementation Science. 2021 Jun 7;16(1):60.
6. Clemson L, Mackenzie L, Roberts C, Poulos R, Tan A, Lovarini M, et al. Integrated solutions for sustainable fall prevention in primary care, the iSOLVE project: a type 2 hybrid effectiveness-implementation design. Implementation Science. 2017 Feb 7;12(1):12.
7. Cushman GK, Koinis-Mitchell D, Alsina M, Barker D, Drew L, DeCesare C, et al. Design of a community-based, Hybrid Type II effectiveness-implementation asthma intervention study: The Rhode Island Asthma Integrated Response (RI-AIR) program. Contemp Clin Trials. 2023 Jun;129:107204.
8. Espel-Huynh HM, Wing RR, Goldstein CM, Thomas JG. Rationale and design for a pragmatic effectiveness-implementation trial of online behavioral obesity treatment in primary care. Contemporary Clinical Trials. 2019 Jul 1;82:9–16.
9. Fasugba O, Dale S, McInnes E, Cadilhac DA, Noetel M, Coughlan K, et al. Evaluating remote facilitation intensity for multi-national translation of nurse-initiated stroke protocols (QASC Australasia): a protocol for a cluster randomised controlled trial. Implementation Science. 2023 Jan 26;18(1):2.
10. Foley KL, Miller DP, Weaver K, Sutfin EL, Petty WJ, Bellinger C, et al. The OaSiS trial: A hybrid type II, national cluster randomized trial to implement smoking cessation during CT screening for lung cancer. Contemporary Clinical Trials. 2020 Apr 1;91:105963.
11. Galaviz KI, Estabrooks PA, Ulloa EJ, Lee RE, Janssen I, López Y Taylor J, et al. Evaluating the effectiveness of physician counseling to promote physical activity in Mexico: an effectiveness-implementation hybrid study. Transl Behav Med. 2017 Dec;7(4):731–40.
12. Garner BR, Gotham HJ, Chaple M, Martino S, Ford JH, Roosa MR, et al. The implementation and sustainment facilitation strategy improved implementation effectiveness and intervention effectiveness: Results from a cluster-randomized, type 2 hybrid trial. Implementation Research and Practice. 2020 Jan 1;1:2633489520948073.
13. Geraci JC, Finley EP, Edwards ER, Frankfurt S, Kurz AS, Kamdar N, et al. Partnered implementation of the veteran sponsorship initiative: protocol for a randomized hybrid type 2 effectiveness—implementation trial. Implementation Science. 2022 Jul 8;17(1):43.
14. Hartzler B, Lyon AR, Walker DD, Matthews L, King KM, McCollister KE. Implementing the teen marijuana check-up in schools—a study protocol. Implementation Science. 2017 Aug 10;12(1):103.
15. Haynes T, Turner J, Smith J, Curran G, Bryant-Moore K, Ounpraseuth ST, et al. Reducing depressive symptoms through behavioral activation in churches: A Hybrid-2 randomized effectiveness-implementation design. Contemporary Clinical Trials. 2018 Jan 1;64:22–9.
16. Iverson KM, Dichter ME, Stolzmann K, Adjognon OL, Lew RA, Bruce LE, et al. Assessing the Veterans Health Administration’s response to intimate partner violence among women: protocol for a randomized hybrid type 2 implementation-effectiveness trial. Implementation Science. 2020 May 7;15(1):29.
17. Lane-Fall MB, Christakos A, Russell GC, Hose BZ, Dauer ED, Greilich PE, et al. Handoffs and transitions in critical care—understanding scalability: study protocol for a multicenter stepped wedge type 2 hybrid effectiveness-implementation trial. Implementation Science. 2021 Jun 15;16(1):63.
18. Lauria ME, Fiori KP, Jones HE, Gbeleou S, Kenkou K, Agoro S, et al. Assessing the Integrated Community-Based Health Systems Strengthening initiative in northern Togo: a pragmatic effectiveness-implementation study protocol. Implementation Science. 2019 Oct 16;14(1):92.
19. Lawson GM, Comly R, Beidas RS, Khanna MS, Goldstein J, Brizzolara-Dove S, et al. Therapist and supervisor perspectives about two train-the-trainer implementation strategies in schools: A qualitative study. Implementation Research and Practice. 2023 Jan 1;4:26334895231190856.
20. Leonard C, Lawrence E, McCreight M, Lippmann B, Kelley L, Mayberry A, et al. Implementation and dissemination of a transition of care program for rural veterans: a controlled before and after study. Implementation Science. 2017 Oct 23;12(1):123.
21. Linendoll N, Murphy-Banks R, Sae-Hau M, Rodday AM, DiFilippo C, Jain A, et al. Evaluating the role of financial navigation in alleviating financial distress among young adults with a history of blood cancer: A hybrid type 2 randomized effectiveness- implementation design. Contemp Clin Trials. 2023 Jan;124:107019.
22. Lyon AR, Pullmann MD, Dorsey S, Levin C, Gaias LM, Brewer SK, et al. Protocol for a hybrid type 2 cluster randomized trial of trauma-focused cognitive behavioral therapy and a pragmatic individual-level implementation strategy. Implementation Science. 2021 Jan 7;16(1):3.
23. Maru S, Nirola I, Thapa A, Thapa P, Kunwar L, Wu WJ, et al. An integrated community health worker intervention in rural Nepal: a type 2 hybrid effectiveness-implementation study protocol. Implementation Science. 2018 Mar 29;13(1):53.
24. McCrimmon T, Gilbert L, Hunt T, Terlikbayeva A, Wu E, Darisheva M, et al. Improving HIV service delivery for people who inject drugs in Kazakhstan: study protocol for the Bridge stepped-wedge trial. Implementation Science. 2019 Jun 14;14(1):62.
25. Neshteruk CD, Skinner AC, Counts J, D’Agostino EM, Frerichs L, Howard J, et al. Translating knowledge into action for child obesity treatment in partnership with Parks and Recreation: study protocol for a hybrid type II trial. Implementation Science. 2023 Feb 24;18(1):6.
26. Owen RR, Woodward EN, Drummond KL, Deen TL, Oliver KA, Petersen NJ, et al. Using implementation facilitation to implement primary care mental health integration via clinical video telehealth in rural clinics: protocol for a hybrid type 2 cluster randomized stepped-wedge design. Implementation Science. 2019 Mar 21;14(1):33.
27. Price-Haywood EG, Burton J, Harden-Barrios J, Bazzano A, Lefante J, Shi L, et al. Depression, anxiety, pain and chronic opioid management in primary care: Type II effectiveness-implementation hybrid stepped wedge cluster randomized trial. Contemporary Clinical Trials. 2021 Feb 1;101:106250.
28. Relph S, Coxon K, Vieira MC, Copas A, Healey A, Alagna A, et al. Effect of the Growth Assessment Protocol on the DEtection of Small for GestatioNal age fetus: process evaluation from the DESiGN cluster randomised trial. Implementation Science. 2022 Sep 5;17(1):60.
29. Reza TF, Nalugwa T, Farr K, Nantale M, Oyuku D, Nakaweesa A, et al. Study protocol: a cluster randomized trial to evaluate the effectiveness and implementation of onsite GeneXpert testing at community health centers in Uganda (XPEL-TB). Implementation Science. 2020 Apr 21;15(1):24.
30. Sanchez A, Pablo S, Garcia-Alvarez A, Dominguez S, Grandes G, Bengoetxea A, et al. Effectiveness of two procedures for deploying a facilitated collaborative modeling implementation strategy—the PVS-PREDIAPS strategy—to optimize type 2 diabetes prevention in primary care: the PREDIAPS cluster randomized hybrid type II implementation trial. Implementation Science. 2021 May 27;16(1):58.
31. Smith JD, Berkel C, Jordan N, Atkins DC, Narayanan SS, Gallo C, et al. An individually tailored family-centered intervention for pediatric obesity in primary care: study protocol of a randomized type II hybrid effectiveness–implementation trial (Raising Healthy Children study). Implementation Science. 2018 Jan 15;13(1):11.
32. Tabak RG, Schwarz CD, Kemner A, Schechtman KB, Steger-May K, Byrth V, et al. Disseminating and implementing a lifestyle-based healthy weight program for mothers in a national organization: a study protocol for a cluster randomized trial. Implementation Science. 2019 Jun 25;14(1):68.
33. Williams CR, Bogdewic S, Owen MD, Srofenyoh EK, Ramaswamy R. A protocol for evaluating a multi-level implementation theory to scale-up obstetric triage in referral hospitals in Ghana. Implementation Science. 2020 May 12;15(1):31.
34. Wolff JM, McQueen A, Garg R, Thompson T, Fu Q, Brown DS, et al. Expanding population-level interventions to help more low-income smokers quit: Study protocol for a randomized controlled trial. Contemp Clin Trials. 2023 Jun;129:107202.
